# Direction of the association between body fatness and self-reported screen time in Dutch adolescents

**DOI:** 10.1186/1479-5868-9-4

**Published:** 2012-01-24

**Authors:** Teatske M Altenburg, Amika S Singh, Willem van Mechelen, Johannes Brug, Mai JM Chinapaw

**Affiliations:** 1 VU University Medical Center, EMGO Institute for Health and Care Research, Department of Public and Occupational Health, Amsterdam, The Netherlands; 2 VU University Medical Center, EMGO Institute for Health and Care Research, Department of Epidemiology & Biostatistics, Amsterdam, The Netherlands

**Keywords:** Adolescents, body fatness, screen time, causality

## Abstract

**Background:**

Screen time has been associated with pediatric overweight. However, it is unclear whether overweight predicts or is predicted by excessive amounts of screen time. The aim of this study was to examine the direction of the association between screen time and body fatness in Dutch adolescents.

**Methods:**

Longitudinal data of 465 Dutch adolescents (mean age at baseline 13 years, 53% boys) was used. Body fatness (objectively measured BMI, four skin folds and waist- and hip circumference), self-reported time spent watching TV and computer use, and aerobic fitness (shuttle run test) were assessed in all participants at three time points during 12 months. Multi-level linear autoregressive analyses was used to examine whether screen time predicted body fatness in the following time period and whether body fatness predicted screen time. Analyses were performed for boys and girls separately and adjusted for ethnicity and aerobic fitness.

**Results:**

Time spent TV viewing did predict changes in BMI and hip circumference in boys, but not in girls, in the subsequent period. Computer time significantly predicted increases in skinfolds in boys and girls and increases in BMI in girls. Body fatness did not predict any changes in screen time.

**Conclusion:**

The present study only partly supports the widely posited hypothesis that higher levels of screen time cause increases in body fatness. In addition, this study demonstrates that high levels of body fatness do not predict increases in screen time.

## Background

The prevalence of overweight and obesity among youth has increased worldwide. Importantly, obesity in youth is known to track into adulthood [1] and is associated with serious health complications such as glucose intolerance, type 2 diabetes and cardiovascular diseases later in life [2]. A 'Westernized' lifestyle of excessive energy intake and sedentary behavior is hypothesized as an important factor in the worldwide increasing prevalence of overweight and obesity [3-7].

A number of cross-sectional and longitudinal studies have demonstrated the positive association between time spent sedentary and measures of body fatness among youth [8-15]. Hume et al [16] showed a dose-response association between screen time and overweight among girls. TV viewing is indicated as the most important sedentary behavior affecting body fatness [17], probably because it is usually clustered with other obesogenic behaviors such as the consumption of soft drinks and high energy snacks [18-20].

Metcalf et al [21] recently questioned the direction of causality between objective measures of physical activity (PA) and pediatric body fatness. Their study demonstrated that body fatness predicted decreased PA, but insufficient PA did not predict increases in body fatness. Importantly, a lack of PA is distinct from sedentary behavior and physiological responses and adaptations to sedentary behavior are not just the opposite of responses to exercise [22]. For example, youth that engage in plenty of PA may also engage in excessive sedentary activities [10]. Ekelund et al [23] demonstrated that levels of TV viewing and PA were independently associated with adiposity among youth. PA and sedentary behavior are not closely inversely related, and these behaviors should therefore be considered as separate entities [23-26], with separate behavioral patterns, determinants and consequences.

In adults, Ekelund et al [27] studied the direction of causality between sedentary behavior and body fatness. They found a reversed causality between sedentary behavior and obesity, indicating that obesity may predict future sedentary time but sedentary time did not predict future obesity. To date, it is still unclear whether body fatness predicts or is predicted by screen time, and no studies among youth have been published on this topic.

Therefore the aim of the present study was to investigate the direction of the association between screen time and body fatness in adolescents. We hypothesized that screen time would predict future body fatness, and that body fatness would also predict future screen time.

## Methods

### Design and participants

The adolescents selected for this study participated in the Dutch Obesity Intervention in Teenagers (DOiT). The DOiT-study is a randomized controlled trial in which the effectiveness of a school-based multi-component intervention aimed at preventing excessive weight gain was evaluated (for details see Singh et al [28]). Data were collected at baseline (September/October 2003; T0) and after eight (T1), twelve (T2) and 20 months (T3). All aspects of the study were approved by the ethics committee of the VU University Medical Center. To minimize confounding effects of the DOiT-intervention, only the adolescents assigned to the control group were included in the current analyses.

Data of 465 adolescents (53% boys) were available for the present analyses (e.g. at T1, T2 and T3). Due to missing data sample sizes varied between 247 and 177 for boys and between 218 and 173 for girls.

### Measurements

#### Body fatness and aerobic fitness

Measures of body fatness included body mass index (BMI), waist circumference (WC), hip circumference (HC) and skinfold thickness (measured at the triceps, biceps, subscapular and suprailiac sites). All measures were performed using a standardized protocol with participants dressed in underwear (for details see Singh et al [28]). Body height (cm) was measured to the nearest 1 mm using a portable stadiometer. Body weight (kg) was measured to the nearest 0.1 kg using a calibrated electronic flat scale. Body height and body weight were measured in order to calculate BMI (kg/m^2^). WC and HC (cm) were measured to the nearest 0.5 cm using a flexible band. Skinfold thickness (mm) was measured to the nearest 0.2 mm using a Harpenden skinfold caliper, and averaging 2 measurements. Values for intrarater reliability (ICC) ranged from 0.82 to 0.95 and values for interrater reliability (ICC) ranged from 0.88 to 0.99 [29]. A group-administrated shuttle run test (adapted 18 m version) was conducted to assess aerobic fitness [30].

#### Screen time

The average time spent watching TV and using the computer (minutes/day; weekdays and weekend days combined), was assessed by self-report [28] comparable to the validated questionnaire of Robinson et al [31]. Total screen time was calculated by summing minutes spent in TV viewing and computer use.

### Statistics

Descriptive subject characteristics (mean (SD)) were calculated at each time point. Differences between boys and girls were examined using the nonparametric Mann-Whitney U test. Since a previous study found that dose-response associations between screen time and overweight were only found among adolescent girls [16], separate analyses were performed for boys and girls. Multi-level linear regression analysis (MLwiN, version 2.22; Centre for Multilevel Modeling) was used to examine whether the amount of screen time predicted changes in measures of body fatness, and whether measures of body fatness predicted changes in the amount of screen time. In these analyses the outcome variable at time point t was related to the value of the predictor at time point t-1, adjusted for the value of the outcome variable at t-1 (autoregressive model). In addition, adjustments were made for ethnicity (based on the country of birth of the parents) and aerobic fitness. The association found can be interpreted as an association of the predictor with changes in the outcome measure, independent of ethnicity and aerobic fitness. Finally, interaction effects for time were checked by including a 'time × predictor' interaction term in the analyses. Using multilevel analyses, regression coefficients can be adjusted for the clustering of observations within one school and/or class. Three levels were defined: 1) student, 2) class, and 3) school. The level of significance was set at P < 0.05.

## Results

Participant characteristics are shown in Table 1. Boys were slightly but significantly older than girls and boys were significantly taller at T1, T2 and T3. At all time points, HC and skinfold thickness were significantly lower for boys and boys had a significantly higher aerobic fitness than girls. At T0, boys spent significantly more time using the computer than girls. Furthermore, at T3 BMI was significantly lower, WC was significantly higher for boys and boys spent significantly more time watching TV than girls.

**Table 1:** Descriptive participant characteristics (Mean ± SD) for Dutch adolescent boys and girls separately.

		T0	T1	T2	T3
**Boys**					

	Age, yrs	12.9 ± 0.5*			
	Ethnicity, %				
	Western	84.3			
	Non-Western	15.7			
	Height, cm	159.7 ± 8.2	163.0 ± 8.6)*	166.4 ± 8.7*	170.8 ± 8.5*
	Weight, kg	49.0 ± 10.1	52.0 ± 10.6)	55.2 ± 11.1	58.8 ± 10.8
	BMI, kg/m^2^	19.1 ± 2.9	19.5 ± 2.9)	19.8 ± 3.0	20.0 ± 2.7*
	Weight status, %				
	Healthy weight	78.9	81.7	80.1	84.0
	Overweight (obese)	19.4 ± 1.7	16.6 ± 1.)	16.7 ± 3.0	14.4 ± 1.7)
	WC, cm	68.1 ± 7.6	69.0 ± 7.2	69.7 ± 7.5	72.8 ± 8.1)*
	HC, cm	82.3 ± 7.4*	84.2 ± 7.6*	87.0 ± 7.5*	86.1 ± 7.0)*
	Skinfold thickness, mm	41.6 ± 20.7*	41.2 ± 21.8*	40.5 ± 21.9*	43.7 ± 22.0)*
	Shuttle run, laps	8.6 ± 2.1*	9.0 ± 2.2*	9.3 ± 2.3*	9.4 ± 2.4)*
	TV viewing, min/day	174.9 ± 97.6	141.6 ± 85.9	141.5 ± 76.8	148.4 ± 83.1)*
	Computer use, min/day	132.6 ± 85.1*	124.7 ± 88.2	132.7 ± 81.3	132.4 ± 89.0)

**Girls**					

	Age, yrs	12.7 ± 0.5)			
	Ethnicity, %				
	Western	89			
	Non-Western	11			
	Height, cm	158.8 ± 6.7	161.4 ± 6.4	163.3 ± 6.1	165.1 ± 5.9
	Weight, kg	49.4 ± 10.7	52.3 ± 11.0	54.2 ± 10.6	57.0 ± 10.8
	BMI, kg/m^2^	19.5 ± 3.4	20.0 ± 3.5	20.3 ± 3.4	20.9 ± 3.6
	Weight status, %				
	Healthy weight	78.2	81.6	80.1	79.8
	Overweight (obese)	17.5 (4.3)	15.5 (2.9)	16.8 (3.1)	16.2 (4.0)
	WC, cm	67.2 ± 8.0	68.9 ± 8.1	68.7 ± 8.0	70.4 ± 8.4
	HC, cm	85.3 ± 8.4	87.9 ± 9.0	89.8 ± 8.8	91.4 ± 8.3
	Skinfold thickness, mm	55.1 ± 27.1	56.0 ± 26.8	54.6 ± 22.4	68.7 ± 28.7
	Shuttle run, laps	7.2 ± 1.9	7.5 ± 1.8	7.6 ± 1.9	7.6 ± 2.0
	TV viewing, min/day	156.4 ± 95.3	134.8 ± 89.8	133.6 ± 73.9	123.5 ± 69.0
	Computer use, min/day	103.8 ± 78.3	119.7 ± 95.4	128.4 ± 88.6	126.0 ± 82.1

* = significantly different between boys and girls. P < 0.002

T0 = Baseline; T1 = 8 months after baseline; T2 = 12 months after baseline; T3 = 20 months after baseline; BMI = body mass index; WC = waist circumference; HC = hip circumference.

### Does body fatness predict screen time?

Body fatness did not predict any changes in TV viewing time and computer time in adolescent boys and girls (Table 2).

**Table 2:** Prospective associations (b (95% CI)) of body fatness on screen time in Dutch adolescents, adjusted for ethnicity and aerobic fitness.

	Boys	Girls
**BMI - b (95% CI)**		

Δ TV viewing	-0.11 (-2.22;1.99)	0.92 (-0.89;2.74)
Δ Computer use	0.60 (-2.05;3.25)	0.68 (-1.58;2.93)

**WC - b (95% CI)**		

Δ TV viewing	0.08 (-0.77;0.92)	0.11 (-0.63;0.86)
Δ Computer use	0.43 (-0.63;1.50)	0.27 (-0.66;1.20)

**HC - b (95% CI)**		

Δ TV viewing	0.33 (-0.43;1.10)	0.35 (-0.36;1.06)
Δ Computer use	0.66 (-0.30;1.61)	0.73 (-0.17;1.63)

**SKINFOLD - b (95% CI)**		

Δ TV viewing	0.05 (-0.26;0.37)	0.01 (-0.24;0.26)
Δ Computer use	-0.13 (-0.53;0.28)	0.01 (-0.35;0.37)

* = significant association between body fatness and changes in screen time

BMI, body mass index, m/kg2; CI, Confidence Interval; Computer use in min/day; HC, hip circumference, cm; Skinfold, skinfold thickness, mm; TV, television viewing, min/day; WC, waist circumference, cm.

### Does screen time predict body fatness?

TV viewing time significantly predicted changes in BMI and HC in boys (Table 3). TV viewing time did not predict changes in body fatness in girls (Table 3). Computer time significantly predicted changes in skinfold thickness in boys and girls and in BMI in boys (Table 3). Furthermore, time significantly modified the association between computer time and skinfold thickness in girls (b = 0.026), indicating that the strength of this association increases in time. These results indicate that when using the computer for 119 min per day (average time spent using the computer for girls in this study; Table 1), the sum of skinfolds may increase with 1.79 cm in girls in a subsequent time period of 2 years (b = 0.015; Table 3).

**Table 3:** Prospective associations (b (95% CI)) of screen time on body fatness in Dutch adolescents, adjusted for ethnicity and aerobic fitness.

	Boys	Girls
**TV viewing - b (95% CI)**		

Δ BMI	0.001 (0.001;0.001)*	0.000 (-0.002;0.002)
Δ WC	0.000 (-0.002;0.002)	0.002 (-0.004;0.004)
Δ HC	0.002 (0.000;0.004)*	0.001 (-0.001;0.003)
Δ Skinfold	0.003 (-0.005;0.011)	-0.006 (-0.018;0.006)

**Computer use - b (95% CI)**		

Δ BMI	0.001 (0.001;0.001)*	0.000 (-0.002;0.002)
Δ WC	0.001 (-0.003;0.005)	0.001 (-0.003;0.005)
Δ HC	0.001 (-0.003;0.005)	-0.002 (-0.004;0.000)
Δ Skinfold	0.008 (0.0005;0.016)*	0.015 (0.003;0.027)*#

* = significant association between screen time and changes in body fatness; # = significant interaction with time

BMI, body mass index, m/kg2; CI, Confidence Interval; Computer use in min/day; HC, hip circumference, cm; Skinfold, skinfold thickness, mm; TV, television viewing, min/day; WC, waist circumference, cm.

## Discussion

This is the first longitudinal exploration of whether body fatness predicts or is predicted by screen time in Dutch adolescents. The results of this study only partly support the widely posited hypothesis that higher levels of screen time cause increases in body fatness. In addition, the results show that high levels of body fatness do not predict increases in screen time.

### Body fatness as a predictor of excessive screen time

This is the first study exploring whether higher levels of body fatness were associated with subsequent changes in screen time in adolescent boys and girls. No evidence was found for a prospective association between indicators of body fatness and screen time, which is in contrast to the findings of Ekelund et al [27] in adults. One explanation for these contrasting results could be the follow-up duration in the study of Ekelund et al [27] was 5.6 years, whereas in our study this was 1 year. This could indicate that body fatness only predicts sedentary behaviour over a relatively long period of time. Another explanation could be a difference in the type of measurement of time spent sedentary. Ekelund et al [27] calculated sedentary time as all minutes below the flex heart rate, which was defined as the mean of the highest resting heart rate and the lowest heart rate while exercising. Using this measure of sedentary time Ekelund et al [27] included all types of sedentary behaviours, whereas in the current study only self reported screen time (i.e. TV viewing and computer use) was included. These different measures of sedentary time could lead to differences in the total amount of screen time, resulting in differences in the associations between body fatness and screen time.

### Body fatness predicted by excessive screen time

The present study showed that TV viewing time predicted changes in two indicators of body fatness (BMI and HC) in adolescent boys, but not in girls. In addition, we showed that computer time predicted changes in two indicators of body fatness (BMI and skinfold) in adolescent boys and one indicator in girls (skinfold).

Our finding that TV viewing time did not predict changes in body fatness among adolescent girls is in agreement with the results of two high quality studies included in a recent review comprising only prospective studies [7,32,33]. In addition, we showed that TV viewing time was related to changes in BMI and HC, but not WC and skinfold thickness in adolescent boys. Skinfold thickness is demonstrated to be a better predictor of body fatness in youth than BMI [34]. Therefore, we conclude that our results did not convincingly demonstrate that TV viewing time predicts body fatness in adolescent boys. This is in contrast to the findings of three high quality studies included in the recent review of Chinapaw et al [7,12,32,35].

The lack of a convincing positive association between TV viewing time and indicators of body fatness in adolescent boys and girls in the present study could indicate that this association is not linear. However, when data for TV viewing were categorized using cut-offs of 2 and 4 hours/day, such a potential prospective association between TV viewing time and indicators of body fatness could not be confirmed.

The present study demonstrated that computer time predicted changes in skinfold thickness among adolescent boys and girls, and changes in BMI among adolescent boys. The differences in the association of computer time with BMI and skinfold thickness are in line with the finding of Nooyens et al [34], demonstrating that skinfold thickness is a better predictor of body fatness in youth than BMI. Based on the large regression coefficients for skinfold thickness and the consistent findings for boys and girls, our findings indicate that computer time predicts increases in body fatness (as measured by skinfold) in adolescents.

To summarize, our results did not convincingly demonstrate that TV viewing time predicts increases in body fatness, whereas computer time did predict increases in body fatness in adolescents. A positive association between sedentary time and body fatness among adolescent boys and girls could therefore only partly be confirmed.

### Strengths and limitations

Strengths include the prospective design and the sample size of the study population. Moreover, the statistics used (autoregressive models) in the current study enabled research into prediction rather than mere association. A study limitation is the reliance on self-reported screen time, which is sensitive to recall bias and socially desirable answers. Although brief self-reported questionnaires have shown to be adequate for group comparisons regarding TV viewing and computer use [36], a misclassification of the amount of screen time could have masked the association between screen time and body fatness in the present study. In addition, adolescents with higher levels of body fatness may have underreported the actual screen time, as also has been observed for food intake. Indeed in the study of Slootmaker et al [37] it was observed that adolescents overrated their physical activity level. Accelerometry is increasingly used as an objective measure of sedentary behaviour; however, it cannot be used to distinguish between TV time and computer time. Another limitation of the present study is the number of statistical tests performed, which implies that the results should be carefully interpreted. Finally, some relevant factors were not included in our statistical analysis (e.g. diet, cigarette smoking, alcohol use), which might have attenuated the associations found.

Adjustments for aerobic fitness were made in order to investigate the prospective association between screen time and body fatness independent of PA. Although a direct and objective measure of PA would have been a better predictor of the time spent in PA, aerobic fitness has shown to be related to time spent in PA in adolescents [38,39].

## Conclusion

The present study is the first exploring whether body fatness predicts or is predicted by screen time among Dutch adolescents. The findings suggest that computer time, and TV viewing time to a lesser extent, predicts increases in body fatness, while body fatness does not predict changes in screen time. These findings imply that it is important to reduce the total amount of computer time in order to prevent increases in body fatness.

## List of abbreviations

BMI: body mass index; DOiT: Dutch Obesity Intervention in Teenagers; ICC: intra- and interrater reliability; SD: standard deviation; TV: television; WC: waist circumference; HC: hip circumference; PA: physical activity.

## Competing interests

The authors declare that they have no competing interests.

## Authors' contributions

TA was involved in the conception and the design of the study, analysis, data interpretation, drafting and manuscript writing. AS was involved in data acquisition, the conception and design of the study, data interpretation and critically revising the manuscript. WM and JB were involved in data interpretation and critically revising the manuscript. MC was involved in conception and design of the study, data interpretation and critically revising the manuscript. All authors read and approved the final manuscript.
